# Comorbid Anxiety and Depression among Pregnant Pakistani Women: Higher Rates, Different Vulnerability Characteristics, and the Role of Perceived Stress

**DOI:** 10.3390/ijerph17197295

**Published:** 2020-10-06

**Authors:** Shahirose Sadrudin Premji, Sharifa Lalani, Kiran Shaikh, Ayesha Mian, Ntonghanwah Forcheh, Aliyah Dosani, Nicole Letourneau, Ilona S. Yim, Shireen Shehzad Bhamani, MiGHT (Maternal-Infant Global Health Team—Collaborators in Research)

**Affiliations:** 1 School of Nursing, Faculty of Health, York University, Toronto, ON M3J 1P3, Canada; nforcheh@yorku.ca (N.F.); MiGHT.CollaboratorsInResearch@gmail.com (M.i.G.H.T.); 2 School of Nursing and Midwifery, Aga Khan University, Karachi 74800, Pakistan; sharifa.lalani@aku.edu (S.L.); kiran.shaikh@aku.edu (K.S.); shireen.shehzad@aku.edu (S.S.B.); 3 Department of Psychiatry, Aga Khan University, Karachi 74800, Pakistan; ayesha.mian@aku.edu; 4 Faculty of Health, Community and Education, School of Nursing and Midwifery, Mount Royal University, Calgary, AB T3E 6K6, Canada; adosani@mtroyal.ca; 5 Department of Community Health Science, Cumming School of Medicine, University of Calgary, Calgary, AB T2N 4N1, Canada; 6 Faculty of Nursing and Cumming School of Medicine (Pediatrics, Psychiatry & Community Health Sciences), University of Calgary, Calgary, AB T2N 1N4, Canada; nicole.letourneau@ucalgary.ca; 7 Department of Psychological Science, University of California Irvine, Irvine, CA 92697, USA; Ilona.yim@uci.edu

**Keywords:** pregnancy, comorbid anxiety and depression, anxiety, depression, perceived stress, adverse childhood experiences, low- and middle-income countries

## Abstract

Anxiety and depression commonly co-occur during pregnancy and may increase risk of poor birth outcomes including preterm birth and low birth weight. Our understanding of rates, patterns, and predictors of comorbid anxiety and depression is hindered given the dearth of literature, particularly in low- and middle-income (LMI) countries. The aim of this study is (1) to explore the prevalence and patterns of comorbid antenatal anxiety and depressive symptoms in the mild-to-severe and moderate-to-severe categories among women in a LMI country like Pakistan and (2) to understand the risk factors for comorbid anxiety and depressive symptoms. Using a prospective cohort design, a diverse sample of 300 pregnant women from four centers of Aga Khan Hospital for Women and Children in Pakistan were enrolled in the study. Comorbid anxiety and depression during pregnancy were high and numerous factors predicted increased likelihood of comorbidity, including: (1) High level of perceived stress at any time point, (2) having 3 or more previous children, and (3) having one or more adverse childhood experiences. These risks were increased if the husband was employed in the private sector. Early identification and treatment of mental health comorbidities may contribute to decreased adverse birth outcomes in LMI countries.

## 1. Introduction

Mental health during pregnancy (e.g., stress, anxiety, pregnancy-related anxiety, and depression) can influence birth outcomes [1] including preterm birth [2,3] and low birth weight [3]. Limited studies examining the role of comorbid anxiety and depression in this literature indicate comorbid anxiety and depression may further increase the risk of preterm birth or low birth weight [4,5]. Studies examining immune system pathways to preterm birth demonstrate the plausibility, as a correlation exists between cytokines such as interleukin-6 and interluken-12 and depression, anxiety and perceived stress measured in the second [6] or third [4] trimester. Furthermore, cytokine levels were higher in women with comorbid anxiety and depression when compared to women with anxiety only [4]. Finally, increases in cytokines were related to level of depression, and in women with severe anxiety, dysregulation in immune system networks triggered or worsened depressive symptoms [4].

Findings from a meta-analysis consisting of 66 studies and 162,120 women from 30 countries, suggest that one in 10 women experience some form of comorbid anxiety and depression [7]. Within the same study, the prevalence of comorbid anxiety and mild-to-severe depressive symptoms during pregnancy was estimated to be 9.5% (95% confidence interval (CI) 7.8–11.2) based on a subset of 25,592 women from 17 studies [7]. The pooled estimate of the prevalence of comorbid anxiety and moderate or severe depressive symptoms was reduced to 6.3% (95% CI 4.8–7.7) based on a total of 22,270 pregnant women from 17 studies [7]. Patterns of comorbid anxiety and mild-to-severe depressive symptoms indicate that the rates were similar in the first trimester: 11.6% (95% CI 9.0–14.2), second trimester: 10.6% (95% CI 7.2–14.0), and third trimester: 9.5% (95% CI 6.1–13.0), respectively [7]. Very few of the studies were found in this meta-analysis to be from low- and middle-income (LMI) countries, yet the rates of comorbid perinatal mental health conditions may be higher in women from LMI countries, owing to their social circumstances [7,8].

Comorbid anxiety and depression during pregnancy have not been extensively studied, but are thought to have multifactorial etiology with individual (e.g., lower education, young age), family (e.g., social relationships), situations (e.g., life events), and community (e.g., having a health card) [7] influences playing important roles. Several studies examining prevalence and risk factors of comorbid anxiety and depression in the post-partum period provide insight into areas of exploration when considering risk factors for comorbidity in the antenatal period. For example, three or more stressors—Emotional (e.g., personal, family or friend with major illness or injury), relationship (e.g., conflict within family member, trouble with alcohol or illicit drugs, or law), traumatic (i.e., physical, emotional, sexual abuse), and/or financial—Resulted in a six-fold increase in comorbid symptoms of anxiety and depression [8]. Maternal age [9], childcare stress [8], perceived support [9], and perceived stress [7] were also associated with, [8] or predicted higher [7] comorbid symptoms of anxiety and depression.

Pregnancy is a time of increased nutrient demands, and low nutrient status may increase prenatal depression and postpartum depression (i.e., perinatal depression) [10,11]. Food insecurity is a real threat for Pakistani pregnant women as Pakistan ranks poorly on a number of indicators suggesting that the people of Pakistan are at extreme risk of food insecurity (ranking 11th on the risk index among 148 countries worldwide) and there are high levels of hunger [12]. Among pregnant South African women, risk factors for food insecurity included depression, suicidal ideation, and having three or more children [13]. Moreover, women with food insecurity or those diagnosed with an anxiety disorder had higher odds of depression [13]. Global literature [10,11] has demonstrated the relationship between nutrient status (of single nutrients) and perinatal depression. Systematic reviews [10,11] have reported inconsistent or no association between concentrations of essential nutrients or compounds (e.g., vitamin B_12_, folate/ferritin, fats and fatty acids) and perinatal depression. Evidence, however, seems to have accumulated to suggest an association between vitamin D and perinatal depression [11].

Although studies allude to similar risk factors for pregnant and postpartum women, Yelland and colleagues [8] advocate that risk factors for comorbidity may be unique for women during the antenatal period. Understanding the antecedents that contribute to women’s mental health during pregnancy, specifically comorbid anxiety and depression, it is imperative to design appropriate and effective psychosocial interventions. For instance, psychosocial interventions that emphasize treatment of comorbid anxiety and depression without considering social determinants of health may be futile in improving women’s mental health during pregnancy [8].

### Research Questions

Given the above, this paper seeks to address the following two research questions: (1) What are the prevalence and patterns of comorbid antenatal anxiety and depression (in the categories of mild-to-severe and moderate-to-severe) among women in a LMI country such as Pakistan? (2) What are the risk factors for comorbid anxiety and depression (mild-to-severe and moderate-to-severe)?

## 2. Materials and Methods

### 2.1. Study Design

A prospective cohort design, over a period of 10 months, enrolled a diverse sample of 300 pregnant women from four centers of Aga Khan Hospital for Women and Children (AKHWC) in Pakistan, including: Hyderabad, Garden, Kharadar, and Karimabad. AKHWC is a university-affiliated teaching hospital with 8000 deliveries per year and offers psychiatric services at each site.

### 2.2. Study Procedures

We utilized purposive sampling to include low-risk women with singleton pregnancies. Hence, women who experienced chronic heart disease, renal disease, or immune deficiency syndrome prior to pregnancy, women diagnosed with any high-risk obstetric complications, or who conceived using artificial reproductive methods, or who were carrying a multiple pregnancy, or carrying a fetus diagnosed with a genetic abnormality were excluded. Women using antidepressants or other psychotropic drugs were also excluded. The nurse in-charge approached eligible pregnant women in the antenatal clinic waiting area and invited them to participate. Those willing to participate were referred to the research assistant who verified eligibility and obtain informed consent. Inclusion criteria comprised: Women who were 18 years or older, with pregnancy between 12–19 weeks’ gestational age (time 1) based on last menstrual period, able to speak Urdu or English, willing to return after 10 weeks (between 22–29 weeks’ gestational age; time 2) for a follow up visit, and who were planning to deliver at the same facility. Women with self-reported health conditions such as hypertension and diabetes mellitus as a result of pregnancy were not excluded, since these comorbidities often develop as result of stress-related multisystem dysregulation in the pathway to preterm birth (PTB) [2]. Written informed consent was obtained from women who were willing to participate in the study. The study was approved by the Ethical Review Committee of the Aga Khan University (ID: 3564-SON-ERC-15) and Conjoint Health Research Ethics Board of University of Calgary (ID: REB15-2372).

### 2.3. Data Collection

Data were collected at two time points during pregnancy: 12–19 weeks’ gestational age (*n* = 300; time 1), and again 10 weeks later at 22–29 weeks’ gestational age (*n* = 282; time 2). Data were collected using the 10-item pregnancy-related anxiety (PRA) scale [14], the 10-item Urdu version of the Edinburgh depression scale (EDS) [15,16,17], the 10-item perceived stress (PS) scale [18], and the 43-item Adverse Childhood (ACE)-International Questionnaire (IQ) [19]. These scales, including their appropriateness (content and face validity), management of translation/backtranslation, and psychometrics are discussed in more detail elsewhere [20]. Briefly, a woman was classified as suffering from anxiety if she answered “very true” to 3 or more of the 10 items on the PRA scale. In addition, information on potential covariates, such as demographics, behavioral (e.g., alcohol use), pre-pregnancy and pregnancy characteristics, and socio-cultural influences, were collected. Approximately 50 min were required to complete all measures.

Consistent with the literature [7], two measures of depression were defined; (1) mild-to-severe depression defined as woman scoring over 9 on the EDS scale, and (2) moderate-to-severe depression if the EDS score was 13 or more. Comorbidity was defined as the co-existence of anxiety and depression at the same point in time.

### 2.4. Data Analysis

#### 2.4.1. Outcome Variables

With two criteria for depression (mild-to-severe depression and moderate-to-severe depression) and data collected at two time points, we computed four indicators that measured comorbidity of anxiety and mild-to-severe depression at time 1 and 2; and comorbidity of anxiety and moderate-to-severe depression at time 1 and 2. We conducted exploratory univariate and bivariate analyses to understand the pattern of comorbidity at time 1 and time 2 and over both time points. We, therefore, aggregated the measure of comorbid anxiety (mild- or moderate-to-severe) as woman experiencing the corresponding comorbidity at any time during pregnancy, that is in weeks 12–19 and/or 22–29 weeks’ gestational age.

#### 2.4.2. Explanatory Variables

Predictors and covariates included socio-demographic (e.g., age, ethnicity), geographic (recruitment site) and socio-economic (e.g., household income) factors, family composition (e.g., number of children), cultural factors (e.g., sex of child given value placed on male child), health habits (e.g., exercise), medications and supplements during pregnancy, level of allostatic load, and perceived stress. Two sub-scales of ACE, namely whether in their childhood parents or guardians understood their problems and worries, and knew how they were spending their free time while they were not at school or work, as well as whether women used street drugs before pregnancy were also included as such experiences (i.e., childhood emotional neglect/trauma and substance use) are known to influence patterns of comorbidity (e.g., depression occurs before anxiety) [21]. Taking “medications”, which has been found to be predictive of preterm birth in the same population [20], was disaggregated into a number of specific indicators: Taking iron supplements (iron forte, fefor vit, folic acid and myfol), vitamins (multi-vitamins, cac 1000, osnate, qalsa) and hormonal supplements (duphaston and cyclogest). In addition, the number of different types of medications (0–3) that each woman was taking was also included in the analysis. For each woman, the perceived stress score at time 1 and 2 was compared, and the larger score taken as their highest perceived stress score during pregnancy (i.e., the most critical level).

#### 2.4.3. Statistical Models for Risk Factors for Comorbid Anxiety and Depressive Symptoms (Mild-to-Severe and Moderate-to-Severe)

The two outcome variables, defined as comorbid anxiety and mild-to-severe depression and comorbid anxiety and moderate-to-severe depression during pregnancy were analyzed separately. In order to investigate question 1, we used Chi-squared test to determine factors that are associated to each outcome variable, and then obtained and compared interval estimates of the prevalence of comorbidity among different categories of significant factors (α = 0.05).

In question 2, we used multiple logistic regression analysis to determine a parsimonious predictive model for risk factors for comorbidity. In view of the large number of explanatory factors relative to sample size, we used conditional forward stepwise criterion with default inclusion and exclusion probabilities of 0.05 and 0.1, respectively, to add the most significant factors to the model, starting from the null model until no factor led to a significant decrease in the likelihood ratio. We further investigated the effect of perceived stress on the final model, by performing conditional regression to determine which risk factors were retained in a model after adjusting for perceived stress.

## 3. Results

Overall, 282 of 300 enrolled women returned for follow up clinic visits, at each of the following sites: 75 (100%) Karimabad, 100 (90.1%) Garden, 39 (88.6%) Kharadar, and 68 (97.1%) Hyderabad. The women were aged between 18–43 years of age with a mean of 26.8 years at time of recruitment. All of them were married, with mean age at first marriage of 22.7 years and range of 16–36 years. Of the 168 (59.6%) women who had previous births, the mean age at first birth was 23.4 years and range of 17–36 years. With respect to education attainment, 23.8% of the women had no formal education, and 22.3%, 35.8% and 18.1% had attained secondary or high school, completed college/university or had a postgraduate degree, respectively.

Out of 38 women who experienced comorbid anxiety and mild-to-severe depression during pregnancy (Table 1), 22 (57.9%) experienced the same condition at time 1, 14 (36.8%) experienced it at time 2, and 2 (5.3%) experienced it at both time periods. Hence, two women experienced comorbid anxiety and mild-severe depression during their entire pregnancy. By comparison, 26 women experienced comorbid anxiety and moderate-to-severe depression during pregnancy, 12 (46.1%) experienced the same condition at time 1, 12 (46.1%) experienced it at time 2, and 2 (7.7%) experienced it at both time periods. Further analysis revealed that these were the same two women among 38 women who experienced comorbid anxiety and mild-to-severe depression during pregnancy.

### 3.1. Prevalence and Patterns of Comorbidity during Pregnancy

The prevalence of anxiety, mild-to-severe and moderate-to-severe depressive symptoms at enrolment were 14.2% (95% CI 10.6–18.8), 23.4 (95% CI 18.8–28.7) and 12.1% (95% CI 8.7–16.4), respectively. By the time of follow up, the prevalence of anxiety had decreased by about half (7.4%; 95% CI 4.9–11.2), while the prevalence of both mild-to-severe depression (28.4%; 95% CI 23.4–33.9) and moderate-to-severe depression (17.7%; 95% CI 13.7–22.6) had increased. However, as seen from the overlap of the 95% CI, none of the changes were statistically significant. The prevalence of comorbid anxiety and mild-to-severe depressive symptoms during pregnancy was 13.5% (95% CI 9.9–18.0). Out of these, 24 (63.2%) experienced the comorbidity at time 1, 16 (42.1%) experienced it at time 2, and only 2 women had comorbid anxiety and mild-to-severe depressive symptoms at both time 1 and 2. Similarly, only the same two women experienced comorbid anxiety and moderate-to-severe depressive symptoms at both time points out of the 26 (9.2%; 95% CI 6.3–13.2) who experienced this comorbidity during pregnancy. The prevalence of comorbid anxiety and moderate-to-severe depressive symptoms was 5.0% (14/282) at both time points. Hence, women who experienced comorbidity at time 1 were generally different from those who experienced comorbidity at time 2. This provided the basis for our choice of creating a measure of comorbid anxiety and depression (mild-to-severe) at any time point during pregnancy.

Women who experienced anxiety at time 1 had significantly higher mean depression scores (10.9; 95% CI 9.2–12.5) at time 2 compared to women without anxiety (6.4; 95% CI 5.7–7.0). This suggests that developing anxiety at time 1, may be a risk factor for developing high depression at time 2. However, after adjusting for depressive symptoms at time 1, the effect of anxiety at time 1 on depressive symptoms later at time 2 was only marginally significant (*p* = 0.055). The mean depression score at time 2 among women with anxiety was 8.4, (95% CI 6.9–10.0) compared to 6.8 (95% CI 6.6–7.4) among women without anxiety.

### 3.2. Association between Covariates and Comorbidity during Pregnancy

As shown in Table 2, only four of the 32 covariates and potential risk factors had statistically significant associations (*p* < 0.05) with both comorbid anxiety and mild-to-severe depressive symptoms and comorbid anxiety and moderate-to-severe depression during pregnancy, namely: Recruitment site, taking iron supplements, ACE level, and food insecurity. The number of supplements was associated with comorbid anxiety and moderate-to-severe depression (*p* = 0.038) but only marginally associated with comorbid anxiety and mild-to-severe depression during pregnancy (*p* = 0.055).

The association between the recruitment site and comorbidity needs to be interpreted cautiously as none of the women in our sample recruited from the Garden site experienced comorbid anxiety and depression (mild or moderate). Women who did not take iron supplements had much higher prevalence of comorbid anxiety and mild (17.8%) or moderate (12.4%) depression compared to those who took supplements (5.2% and 3.1%). ACE-IQ had a positive association with comorbidity, although the highest prevalence of each indicator of comorbidity was among women with one ACE and the lowest was among women with no ACEs. Women with two or more ACEs had similar prevalence to women with one ACE. While one third (33.3%) of women without food security experienced comorbid anxiety and depression (both mild and moderate), only 8.1% of women with food insecurity experienced comorbid anxiety and moderate-to-severe depressive symptoms. Women who took a combination of supplements also had much lower prevalence of comorbid anxiety and depression (mild-to-severe and moderate-to-severe) than women who took only one supplement or no supplements at all. None of the other socio-demographic covariates were associated with comorbidity.

### 3.3. Risk Factors for Comorbid Anxiety and Mild-to-Severe Depressive Symptoms

Employing a multiple logistic regression model using forward conditional likelihood, only recruitment site (*p* = 0.007) and husband’s employer (*p* = 0.056) were retained as predictors of comorbid anxiety and mild-to-severe depressive symptoms out of the 32 covariates and potential risk factors considered. When perceived stress was included in the model, only husband’s employer was retained as a predictor (*p* = 0.038). Table 3 shows the most parsimonious predictive model for comorbid anxiety and mild-to-severe depressive symptoms. The odds of having comorbid anxiety and mild-to-severe depressive symptoms increased by 1.3 (95% CI 1.17–1.36) times per unit increase in perceived stress score. The odds of having comorbid anxiety and mild-to-severe depressive symptoms were 17.6 times higher if the husband worked in the private sector, and 20.6 times higher if the husband worked for the government when compared to elsewhere. However, the confidence intervals for the odds ratios were very wide, indicating uncertainty regarding precise estimate of the odds.

We compared the goodness of fit of the model for comorbidity of anxiety and mild-to-severe depressive symptoms with and without perceived stress. We found that none (0.0%) of the 39 women experiencing comorbid anxiety and mild-to-severe depressive symptoms were correctly predicted as such using the model without perceived stress, as compared to 7 (17.9%) who were correctly predicted as having experienced comorbidity. The increase in perceived stress during pregnancy is identified as the only risk factor of comorbid anxiety and mild-to-severe depressive symptoms during pregnancy while the sector in which the husband worked is a potential covariate.

### 3.4. Risk Factors for Comorbid Anxiety and Moderate-to-Severe Depressive Symptoms

A different pattern of association emerged when comorbid anxiety and moderate-to-severe depressive symptoms were considered. Without adjusting for perceived stress, recruitment site (*p* = 0.077), ACE-IQ (*p* = 0.006) and food insecurity (*p* = 0.034) were the only variables retained as predictors of comorbid anxiety and moderate-to-severe depressive symptoms during pregnancy. Perceived stress was found to be a strong predictor of comorbidity (*p* < 0.001). As shown in Table 4, the inclusion of perceived stress altered the set of factors retained as risk factors for comorbid anxiety and moderate-to-severe depressive symptoms during pregnancy.

With perceived stress in the model, recruitment site and food insecurity did not emerge as significant predictors, and instead, having parity of more than 3 children (*p* = 0.016), the sector in which the husband worked (*p* = 0.012), and ACE-IQ (0.014) were added as predictors. The odds of a women who had 3 or more previous children having comorbid anxiety and moderate-to-severe depressive symptoms was up to 20 times (19.6; 95% CI 1.7–222.7) that of a woman with less than 3 children ever born. Women whose husbands worked in the private sector also had much higher odds (24.2, 95% CI 2.3–250.3) of experiencing comorbidity than those whose husbands worked in “other” sectors. Women who had experienced one ACE had the highest odds (8.0, 95% CI 2.0–32.1) of comorbidity relative to women with no ACEs.

We found that only one (3.9%) of the 37 women experiencing comorbid anxiety and moderate-to-severe depression was correctly predicted as such using the model without perceived stress, compared with 11 (40.7%) of the women who were correctly predicted as experiencing comorbid anxiety and depression using the model adjusted for perceived stress. Among all the models considered, this was the highest probability of correct classification of women experiencing comorbid anxiety and depression. Overall, 92.6% of women were correctly classified with this model.

## 4. Discussion

Our findings reveal that comorbid anxiety and depression at each time was high and that the greatest risk factors for comorbid anxiety and moderate-to-severe depression during pregnancy were: (1) High level of perceived stress at any time point, (2) having 3 or more previous children, and (3) having one or more ACEs. These risks were increased if the husband worked in the private sector. Growing up with parents or guardians who did not understand their childhood problems and worries appeared to be the most important dimension of ACE in predicting comorbid anxiety and moderate-to-severe depression, although the aggregate measure based on all ACE indicators provided a better model fit than individual components.

Our point estimates of the prevalence of comorbid anxiety and mild-to-severe depression (13.5%) and moderate-to-severe depression (9.2%) were higher than the corresponding rates of 9.5% and 6.3%, respectively, reported in the systematic review with studies predominantly from high-income countries [7]. Prevalence of comorbidity is influenced by geographic location and cultural milieu [22,23]. Differences in prevalence of comorbidity between a cohort of pregnant Spanish (26.9%) and Turkish (47%) women were attributed to sociocultural characteristics and practices including number of living children, quality of relationship with husband, family structure such as extended family living in the household, and ability to work given educational preparation [22,23].

The prevalence and course of comorbid anxiety and depressive symptoms varies across pregnancy (i.e., between first and third trimester) with comorbidity declining between the first and third trimesters [7]. Additional findings in our cohort suggest that there was little overlap between women who experienced comorbidity at 22–29 weeks’ gestational age and those who had experienced corresponding comorbidity at 12–19 weeks’ gestational age. This is a unique finding indicating that there may be different vulnerability characteristics of women who get comorbid anxiety and depressive symptoms at different times of pregnancy. Pregnancy is a socially constructed state [24] with culturally entrenched preferences for male children, elevating some women’s status in their homes and community [25,26]. For the husband and his family, the wife/daughter-in-law suddenly transforms into the mother of the child/children who will propagate the family line and be a source of income for family, especially if she is carrying a male child [25]. Women’s childhood experiences of being disadvantaged given their sex at birth influences personal preferences for a male child [26].

Anxiety disorders often present before depressive symptoms; however, anxiety preceding depression versus depression preceding anxiety, likely have different etiologic pathways [7]. Anxiety is often a salient symptom in a primary depressive disorder. However, there is a distinct probability of having anxiety symptoms and/or disorders (e.g., obsessive-compulsive disorder (OCD), generalized anxiety) without comorbid depressive symptomatology. From a practice perspective, clinicians should not assume that just because a woman is not experiencing comorbidity at a given time she has not experienced comorbidity at an earlier time or would not experience it in the remaining weeks of pregnancy leading up to the delivery.

Perceived stress was found to be a strong predictor of comorbid anxiety and mild-to-severe and moderate-to-severe depressive symptoms. For example, a one unit increase in perceived stress led to a 1.3-fold increase in the odds of having comorbid anxiety and mild-to-severe depressive symptoms. Perceived stress has also been identified as a risk factor of comorbid postpartum depressive symptomatology and anxiety [7]. After adjusting for level of stress, odds of a woman who had 3 or more children having comorbid conditions was up to 26 times that of a woman with less than 3. Inadequate social support or help, as determined by self-report of amount and frequency of practical assistance from family and friends, was a correlate to comorbid anxiety and depression in postpartum Australian women enrolled in a community-based study [27]. Hence, women’s perspective on the practical nature of the amount and frequency of support [27] and help received may add a layer of stress which may lead to symptoms of anxiety and/or depression.

Women whose husbands worked in the private sector also had much higher odds of experiencing comorbidity than those whose husbands worked in another sector, and marginally higher than women who worked for the government. Most government jobs in Pakistan provide a stable income and flexible work hours, which reduces the level of stress and anxiety, as compared to private jobs which are often characterized by long working hours, creating extra pressure [28]. A women’s spouse working in the private sector may act as a source of extra burden on the household, by often transmitting their work stress onto their wives. The husbands would also be less available to provide social support or help to the mother, thereby increasing stress, anxiety, and/or depression.

Women who took a combination of supplements also had much lower prevalence of comorbidity than women who took only one supplement or no supplements at all. A plausible explanation within the sociocultural environment of Pakistan is that women may want or expect to receive medication treatment during their engagement with physicians [29]. Both prescription and non-prescription medication when personalized to the context of women can influence physical and emotional well-being [30]. Although the recruitment site was included as a predictor, its inclusion lead to unstable estimates as evident from the wide confidence intervals. The wide confidence intervals were the result of the finding that none of the 100 women seen in the Garden site had evidence of comorbidity. Team members’ personal reflections and anecdotal experience suggests that women in the Garden site may have had an increased social support network of and from women, as well more opportunity and space for rest and relaxation. Low social support has been identified as a risk factor for anxiety and depression [31]. For this pilot we do not have sufficient statistical power to examine the interactive relationship between social support and comorbid anxiety and depression; however, it would be an important determinant to keep in mind for future studies.

Our findings are consistent with findings from both a low- and middle-income country (Kenya) [32] and a high-income country (USA) which showed an association between ACEs and depression/anxiety and perceived stress [33,34]. Women who endorsed that their own parents/guardians did not understand their childhood problems and worries also had slightly higher odds of comorbidity than those who endorsed that their parents/guardian understood their problems and worries. Using this model, a much higher percentage of women’s comorbid conditions was correctly classified as such, and overall, 93% of women were correctly classified. Although individual ACEs, such as parents/guardians not understanding the child’s problems and worries are associated with psychosocial distress in young adults (early 20’s), an interactive effects is present with perceived life stress [35]. This may explain why ACEs remained significant when perceived stress was included in the model and recruitment site and food insecurity became conditionally insignificant.

Early identification of stress, anxiety and depression in pregnancy will permit the medical and nursing clinicians to implement measures to reduce anxiety and depression and its potential negative consequences such as preterm birth [20]. Limited studies have examined comorbidity risk factors and their etiological pathways in pregnant women. These phenomena need to be explored in a larger study with adequate sample size. Furthermore, it is imperative for researchers to address perinatal mental health in low- and middle-income countries.

Our findings are based on a small sample of Pakistani women predominantly recruited from urban sites and findings and therefore, cannot be generalized to all Pakistani pregnant women. Furthermore, the 10-week time frame in which stress, anxiety, and depression were measured provides a limited assessment of the psychosocial health of these pregnant women throughout the pregnancy. Our study did not determine if women experienced gender disadvantage. Finally, our study used self-report instruments that measured symptoms and are not gold standard diagnostic interviews.

## 5. Conclusions

Comorbid anxiety and depression is an important public health concern as it suggests an increased vulnerability to social determinants of negative health outcomes and relies on the capacity of pregnant women to draw from social support networks to mitigate these risks. Essentially, ACEs and perceived stress stand out as the primary predictors of comorbidity, and various other factors provide interesting insights into patterns of comorbidity at different time points during pregnancy. Comorbidity signals poor psychological well-being, poor coping resources, and threatens adverse pregnancy and infant outcomes. Nurses, midwives and medical clinicians should integrate mental health assessments of every women across pregnancy as a standard of care as women who experience comorbidity early in pregnancy in order to capture both this group of women, and the largely separate group who experience comorbidity during late second trimester.

## Figures and Tables

**Table 1:** Comorbid anxiety and depressive (mild-to-severe and moderate-to-severe) symptoms between 12–19 weeks’ gestational age (time 1) at follow-up after 10 weeks (between 22–29 weeks’ gestational age; time 2).

Symptom(s)	Gestational Period (n = 282)			
	Time 1 (12–19 wks)	Time 2 (22–29 wks)	Both Time 1 & 2	Any Time
	*n* (%, 95% CI)	*n* (%, 95% CI)	n (%, 95% CI)	*n* (%, 95% CI)
Anxiety	40 (14.2, 10.6–18.8)	21 (7.4, 4.9–11.2)	6 (2.8, 0.9–4.7)	55 (19.9, 15.3–24.5)
Mild-to-Severe Depression	66 (23.4, 18.8–28.7)	80 (28.4, 23.4–33.9)	43 (15.7, 11.5–19.9)	103 (36.7, 31.1–42.3)
Comorbid Anxiety and Mild-to-Severe Depression	24 (9.1, 5.7–12.4)	16 (6.3, 3.5–9.1)	2 (1.4, 0.0–2.7)	38 (13.5, 9.9–18.0)
Moderate-to-Severe Depression	34 (12.1, 8.7–16.4)	50 (17.7, 13.7–22.6)	15 (5.9, 3.2–8.7)	69 (24.8, 19.8–29.8)
Comorbid Anxiety and Moderate-to-Severe Depression	14 (5.6, 2.9–8.2)	14 (5.6, 2.9–8.2)	2 (1.4, 0.0–2.7)	26 (9.8, 6.3–13.2)

Note. wks = weeks; CI = confidence interval.

**Table 2:** Factors associated with comorbid anxiety and depression during pregnancy.

	Factors	Comorbid Anxiety and		
		Mild-to-Severe Depressive Symptoms	Moderate-to-Severe Depressive Symptoms	
		*χ^2^ (p-value)*	*χ^2^ (p-value)*	df
1	Recruitment Site	**24.5 (0.001)**	**16.2 (0.001)**	3
2	Iron supplements	**8.8 (0.003)**	**6.6 (0.010)**	1
3	ACE level	**7.4 (0.025)**	**12.6 (0.002)**	1
4	Food insecurity	**4.2 (0.040)**	**8.7 (0.003)**	1
5	Number of supplements	5.8 (0.055)	**6.5 (0.038)**	1

χ^2^ = Chi-squared; df = degrees of freedom; ACE = adverse childhood experiences.

**Table 3:** Odds ratio and 95% confidence interval of predictors of comorbid anxiety and mild-to-severe depression.

Variables in Equation	$\mathrm{Parameter}\;\mathrm{Estimate}\;\left(\hat{\beta }\right)\;$	*p*-Value	Odds Ratio (OR)	95% CI for OR	
				Lower	Upper
**Perceived stress**	0.234	0.000	1.3	1.17	1.36
**Husband’s employer**		0.038			
Private	2.868	0.011	17.6	1.9	162.5
Government	3.026	0.019	20.6	1.7	256.6
Other (reference)	0.000		1.0		
Constant	0.241	0.000	0.000		

**Table 4:** Odds ratio and 95% confidence interval of comorbid anxiety and moderate-to-severe depression.

Variables in the Equation	$\mathrm{Parameter}\;\mathrm{Estimate}\;\left(\hat{\beta }\right)$	*p*-Value	Odds Ratio (OR)	95% CI for OR	
				Lower	Upper
**Highest Perceived Stress**	0.263	<0.001	1.3	1.2	1.4
**More than 3 CEB**		0.016			
Yes	2.975	0.016	19.6	1.7	222.7
No (reference)	0.000		1.0		
**Husband’s employer**		0.012			
Private	3.188	0.007	24.2	2.3	250.3
Government	0.709	0.681	2.0	0.07	59.8
Other (reference)	0.000		1.0		
**ACE-IQ Binary**		0.014			
None (reference)	0.000		1.0		
One	2.078	0.003	8.0	2.0	32.1
Two or more	1.478	0.040	4.4	1.1	17.9
Constant	−12.024	0.000			

CEB = children ever born; ACE-IQ = adverse childhood experiences-international questionnaire.

## References

[1] Satyanarayana V.A., Lukose A., Srinivasan K. (2011). Maternal mental health in pregnancy and child behavior. Indian J. Psychiatry.

[2] Premji S. (2014). Perinatal distress in women in low-and middle-income countries: Allostatic load as a framework to examine the effect of perinatal distress on preterm birth and infant health. Matern. Child Health J..

[3] Buffa G., Dahan S., Sinclair I., St-Pierre M., Roofigari N., Mutran D., Rondeau J.-J., Dancause K.N. (2018). Prenatal stress and child development: A scoping review of research in low-and middle-income countries. PLoS ONE.

[4] Leff-Gelman P., Mancilla-Herrera I., Flores-Ramos M., Saravia-Takashima M.F., Cruz-Coronel F.M., Cruz-Fuentes C., Pérez-Molina A., Hernández-Ruiz J., Silva-Aguilera F.S., Farfan-Labonne B. (2019). The cytokine profile of women with severe anxiety and depression during pregnancy. Bmc Psychiatry.

[5] Uguz F., Yakut E., Aydogan S., Bayman M.G., Gezginc K. (2019). The impact of maternal major depression, anxiety disorders and their comorbidities on gestational age, birth weight, preterm birth and low birth weight in newborns. J. Affect. Disord..

[6] Latendresse G., Ruiz R.J., Wong B. (2013). Psychological distress and SSRI use predict variation in inflammatory cytokines during pregnancy. Open J. Obstet. Gynecol..

[7] Falah-Hassani K., Shiri R., Dennis C.L. (2017). The prevalence of antenatal and postnatal co-morbid anxiety and depression: A meta-analysis. Psychol. Med..

[8] Yelland J., Sutherland G., Brown S.J. (2010). Postpartum anxiety, depression and social health: Findings from a population-based survey of Australian women. Bmc Public Health.

[9] Dennis C.L., Brown H.K., Wanigaratne S., Vigod S.N., Grigoriadis S., Fung K., Marini F., Brennenstuhl S. (2018). Determinants of comorbid depression and anxiety postnatally: A longitudinal cohort study of Chinese-Canadian women. J. Affect. Disord..

[10] Trujillo J., Vieira M.C., Lepsch J., Rebelo F., Poston L., Pasupathy D., Kac G. (2018). A systematic review of the associations between maternal nutritional biomarkers and depression and/or anxiety during pregnancy and postpartum. J. Affect. Disord..

[11] Sparling T.M., Nesbitt R.C., Henschke N., Gabrysch S. (2017). Nutrients and perinatal depression: A systematic review. J. Nutr. Sci..

[12] Khan M.A., Akhtar-Ali-Shah S. (2011). Food Insecurity in Pakistan: Causes and Policy Response. J. Agric. Environ. Ethics.

[13] Abrahams Z., Lund C., Field S., Honikman S. (2018). Factors associated with household food insecurity and depression in pregnant South African women from a low socio-economic setting: A cross-sectional study. Soc. Psychiatry Psychiatr. Epidemiol..

[14] Rini C.K., Dunkel-Schetter C., Wadhwa P.D., Sandman C.A. (1999). Psychological adaptation and birth outcomes: The role of personal resources, stress, and sociocultural context in pregnancy. Health Psychol..

[15] Cox J.L., Holden J.M., Sagovsky R. (1987). Detection of postnatal depression. Development of the 10-item Edinburgh Postnatal Depression Scale. Br. J. Psychiatry.

[16] Bergink V., Kooistra L., Lambregtse-van den Berg M.P., Wijnen H., Bunevicius R., van Baar A., Pop V. (2011). Validation of the Edinburgh Depression Scale during pregnancy. J. Psychosom. Res..

[17] Cox J.L., Chapman G., Murray D., Jones P. (1996). Validation of the Edinburgh postnatal depression scale (EPDS) in non-postnatal women. J. Affect. Disord..

[18] Cohen S., Kamarck T., Mermelstein R. (1983). A global measure of perceived stress. J Health Soc Behav.

[19] World Health Organization Adverse Childhood Experiences International Questionnaire (ACE-IQ). http://www.who.int/violence_injury_prevention/violence/activities/adverse_childhood_experiences/en/.

[20] Shaikh K., Premji S.S., Lalani S., Forcheh N., Dosani A., Yim I.S., Samia P., Naugler C., Letourneau N. (2020). Ethnic disparity and exposure to supplements rather than adverse childhood experiences linked to preterm birth in Pakistani women. J. Affect. Disord..

[21] de Graaf R., Bijl R.V., Smit F., Vollebergh W.A., Spijker J. (2002). Risk factors for 12-month comorbidity of mood, anxiety, and substance use disorders: Findings from the Netherlands Mental Health Survey and Incidence Study. Am. J. Psychiatry.

[22] González-Mesa E., Kabukcuoglu K., Blasco M., Körükcü O., Ibrahim N., González-Cazorla A., Cazorla O. (2020). Comorbid anxiety and depression (CAD) at early stages of the pregnancy. A multicultural cross-sectional study. J. Affect. Disord..

[23] González-Mesa E., Kabukcuoglu K., Körükcü O., Blasco M., Ibrahim N., Kavas T. (2018). Cultural factors influencing antenatal depression: A cross-sectional study in a cohort of Turkish and Spanish women at the beginning of the pregnancy. J. Affect. Disord..

[24] Sutherland S. (1997). Pregnancy: A Social Construction.

[25] Shukar-Ud-Din S., Ubaid F., Shahani E., Saleh F. (2013). Reasons for disclosure of gender to pregnant women during prenatal ultrasonography. Int. J. Women’s Health.

[26] Qadir F., Khan M.M., Medhin G., Prince M. (2011). Male gender preference, female gender disadvantage as risk factors for psychological morbidity in Pakistani women of childbearing age-a life course perspective. BMC Public Health.

[27] Ramakrishna S., Cooklin A.R., Leach L.S. (2019). Comorbid anxiety and depression: A community-based study examining symptomology and correlates during the postpartum period. J. Reprod. Infant Psychol..

[28] Ahmed S., Jiskani S., Bhatti K. (2011). Measuring Job Satisfaction of Government Sector Employees; A case of Bureau of Statistics, Pakistan. J. Manag. Soc. Sci..

[29] Britten N., Jenkins L., Barber N., Bradley C., Stevenson F. (2003). Developing a measure for the appropriateness of prescribing in general practice. Qual. Saf. Health Care.

[30] Gray N.J., Wood D.M. (2017). The Role of Medication in Supporting Emotional Wellbeing in Young People with Long-Term Needs. Healthcare.

[31] Lee A.M., Lam S.K., Sze Mun Lau S.M., Chong C.S., Chui H.W., Fong D.Y. (2007). Prevalence, course, and risk factors for antenatal anxiety and depression. Obs. Gynecol.

[32] Samia P., Premji S., Tavangar F., Yim I.S., Wanyonyi S., Merali M., Musana W., Omuse G., Forcheh N., Dosani A. (2020). Adverse Childhood Experiences and Changing Levels of Psychosocial Distress Scores across Pregnancy in Kenyan Women. Int. J. Environ. Res. Public Health.

[33] Smith M.V., Gotman N., Yonkers K.A. (2016). Early Childhood Adversity and Pregnancy Outcomes. Matern. Child Health J..

[34] Bowers K., Ding L., Gregory S., Yolton K., Ji H., Meyer J., Ammerman R.T., van Ginkel J., Folger A. (2018). Maternal distress and hair cortisol in pregnancy among women with elevated adverse childhood experiences. Psychoneuroendocrinology.

[35] Manyema M., Norris S.A., Richter L.M. (2018). Stress begets stress: The association of adverse childhood experiences with psychological distress in the presence of adult life stress. BMC Public Health.

